# A National Web-GIS Dashboard to Support African Swine Fever Surveillance in Domestic Pigs and Wild Boars in Italy

**DOI:** 10.3390/pathogens15090925

**Published:** 2026-09-02

**Authors:** Marta Cresci, Daria Di Sabatino, Susanna Tora, Angelo Cerella, Federica Di Gianvito, Carmen Iscaro, Francesco Feliziani, Marco Sordilli, Alessio Di Lorenzo

**Affiliations:** 1 Istituto Zooprofilattico Sperimentale dell’Abruzzo e del Molise “G. Caporale”, Campo Boario, 64100 Teramo, Italy; 2 National Reference Laboratory for Pestivirus and Asfivirus, Istituto Zooprofilattico Sperimentale dell’Umbria e delle Marche “Togo Rosati”, 06126 Perugia, Italy; 3 Ufficio 3—Sanità Animale, Direzione Operativa del Centro Nazionale di Lotta ed Emergenza Contro le Malattie Animali e Sistema I&R, Direzione Generale della Salute Animale, Ministero della Salute, Viale Giorgio Ribotta, 5, 00144 Roma, Italy

**Keywords:** African swine fever, disease control, Geographic Information Systems, Web-GIS, Italy

## Abstract

African swine fever is a fatal disease that affects members of the Suidae family and also threatens human economic activity. Genotype II of the African swine fever virus was detected in the wild boar population in mainland Italy in 2022. Subsequently, the infection spread to domestic pig populations. To support the monitoring of the national surveillance plan and the implementation of control measures, a dedicated African Swine Fever Web-GIS dashboard (ASF Web-GIS dashboard) was developed by the Istituto Zooprofilattico Sperimentale dell’Abruzzo e del Molise “G. Caporale” (IZSAM), in collaboration with the National Reference Centre for the Study of Pestivirus and Asfivirus Diseases (CEREP) and the General Directorate for Animal Health of the Italian Ministry of Health. The ASF Web-GIS dashboard is based on Esri ArcGIS Dashboards and enables users to visualise information collected through Italy’s national information systems, including data related to the surveillance plan, notified outbreaks, restricted areas, and physical barriers established to control disease spread. This dashboard is a useful tool supporting decision-makers in visualising nationwide activities carried out in support of African swine fever control measures. The ASF Web-GIS dashboard is not publicly accessible since it is intended to support ASF monitoring for Veterinary Services, Istituti Zooprofilattici Sperimentali staff, and the General Directorate for Animal Health of the Ministry of Health.

## 1. Introduction

The sole species belonging to the family *Asfarviridae* and genus *Asfivirus* is *Asfivirus haemorrhagiae*, previously named African Swine Fever Virus (ASFV) [1]. ASFV is the etiological agent of African Swine Fever (ASF), a fatal disease affecting members of the family *Suidae*, and representing a serious threat to animal health as the availability of approved commercial vaccines remains limited, with their use in the field currently authorised in Vietnam [2]. Although ASF does not pose a direct risk to human health, it has a notable economic impact [3,4]. In particular, the economic repercussions of ASF on affected areas are largely attributable to the implementation of control measures in domestic and wild *Suidae* populations, including the stamping-out of infected holdings and movement restrictions affecting pigs and pig-derived products [5].

Since 2007, African swine fever virus (ASFV) genotype II has progressively spread from the Caucasus region across Eastern Europe, West Asia, Northern Europe, and Central Europe, affecting both domestic pigs and wild boars [3,6,7]. In parallel, since 2018, the virus emerged in several Asian countries [8], and by 2019–2020, it had further expanded throughout Europe, highlighting its rapid transcontinental spread and persistent threat to swine populations [9]. As of August 2026, 18 European countries have reported ASF infections [10,11].

For the first time in mainland Italy, genotype II of the ASF virus was detected within the wild boar population in the Piedmont region in 2022. Subsequently, additional wild boar carcasses testing positive were detected in the neighbouring Liguria Region [12]. By 2024, the number of affected regions in Italy increased from two to eight (Piedmont, Liguria, Lombardy, Emilia-Romagna, Tuscany, Lazio, Calabria, and Campania), with a cumulative total of more than four thousand cases detected in wild boar and 52 outbreaks in domestic pigs caused by ASFV genotype II by August 2026 [13]. The latter were located in Tuscany, Piedmont, Lombardy, Emilia-Romagna, Lazio and Calabria [13]. Although the disease was successfully eradicated in Lazio, Campania, and Calabria [14,15], the virus continues to spread gradually in north-western Italy. In August 2026, new ASF cases were detected in wild boar in the Lazio region, in an area close to the area previously affected by the disease [13].

Since 2020, in response to the ongoing ASFV epidemic in Europe, a national surveillance plan was implemented in Italy. As recommended by European legislation and based on the available evidence [16], a standardised data collection system was implemented within the Italian National Veterinary Information System (https://www.vetinfo.it/, accessed on 28 August 2026).

The surveillance plan is based on collecting and testing samples from found dead animals and on the notification of every suspect case, in wild boar and domestic pigs. Regarding wild boars, any found dead animal, including road-killed, is reported, sampled and tested for ASF. In affected zones, passive surveillance is enhanced through active searches for wild boar carcasses, based on a 1 km^2^ grid within restricted zones, supported by scent-detection dogs trained for carcass detection. Relating to domestic pigs, sampling for passive surveillance is planned for all suspect cases and also for at least two dead pigs per region per week, primarily on commercial farms with no more than 50 animals or backyard farms. In addition, particularly in restricted zones II and III, the first two pigs that weigh more than 60 kg dead must be sampled on a weekly basis on fattening farms, while on breeding farms, all dead boars and sows must be sampled.

Control measures were applied in compliance with Implementing Regulation (EU) 2021/605 and subsequently amended by Commission Implementing Regulation (EU) 2023/594 [17,18], including the establishment of animal movement restricted zones, enhanced surveillance, and reinforced biosecurity in domestic pig holdings. In particular, to manage the epidemiological situation, Commission Implementing Regulation (EU) 2023/594 [17] requires the establishment of specific restricted zones:

- Restricted zone I: High-risk areas that do not have active cases, but border restricted zones II or III.
- Restricted zone II: Areas where ASF is present strictly in wild boars.
- Restricted zone III: Areas where ASF is present in domestic pigs, with or without wild boar cases.

Containment of infected wild boar populations was pursued through the installation of physical barriers, including the strengthening of those already existing along motorways.

To mitigate the spread of ASFV in wild boar populations, hunting and wildlife control strategies were also implemented, including a zoning approach according to the EU strategy against ASF in wild boar populations [19]. In ASF-free areas, the “National Action Plan for the Capture, Culling, and Disposal of Wild Boar Carcasses in ASF-free Territories” was implemented [20]. This plan aimed to improve hunting and population control efforts, adapting the hunting intensity to the ecological and epidemiological characteristics of each region. Conversely, within restricted zones (I, II, and III), wild boar hunting was prohibited, and population control activities were strictly regulated to prevent disturbance-driven dispersal of ASFV. However, derogations could be granted following prior evaluation and approval by a National Expert Group, established to scientifically support the Italian Veterinary Authority.

**Figure 1:**
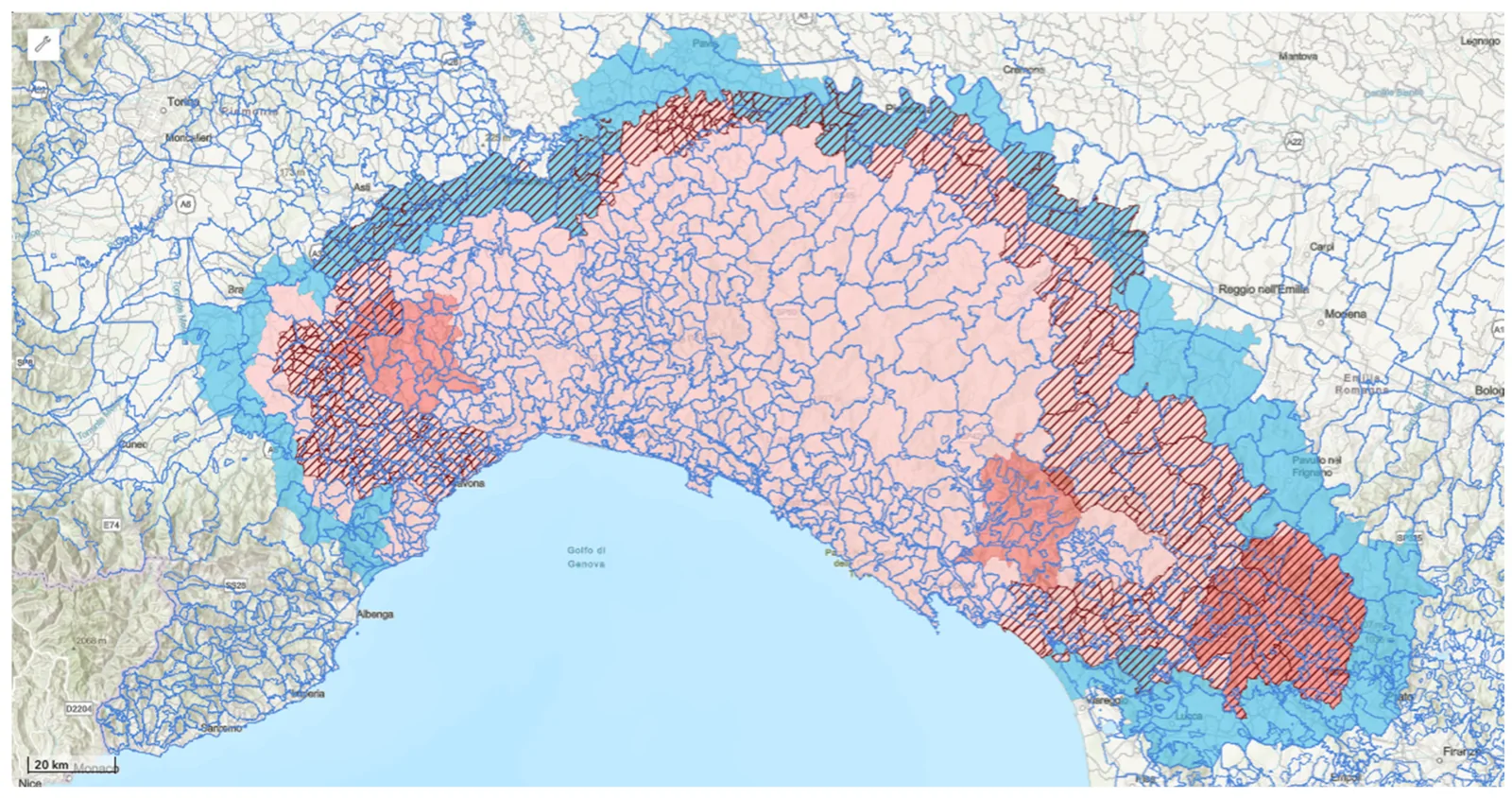
Visualisation of the African Swine Fever (ASF) zoning system implemented in Italy and displayed in the ASF Web-GIS dashboard. The blue perimeter delineates the Italian hunting grounds. Areas shaded in light blue correspond to ASF restricted zone I, pink areas represent ASF restricted zone II, and red areas indicate ASF restricted zone III. The hatched area denotes the CEV zone. Source: ASF Web-GIS dashboard, accessed on 19 August 2026.

In 2025, a Viral Expansion Control Zone (CEV Zone) was defined [21]; the CEV Zone is a high-risk area for disease transmission, which overlaps partially with restricted zones I and II, where specific activities and measures must be carried out to assess the true extent of the infection and prevent its spread. An additional wild boar density reduction zone of about 20 km was established, extending from the outer boundary of the CEV Zone or restricted zone I, into a disease-free area [21]. This zone is designated for the implementation of intensive depopulation and wild boar control activities, which are carried out by the UGC (Unità di Gestione del Cinghiale). The Italian zoning system for African Swine Fever is displayed in Figure 1.

All these activities generated epidemiological and operational data, which were systematically collected to support disease control efforts and structured to ensure compliance with reporting obligations to the European Food Safety Authority [22], the European Commission [23], and the World Organisation for Animal Health (WOAH) [24].

Immediately following the confirmation of the first ASF case in 2022, the need arose for a near-real-time visualisation tool capable of integrating surveillance and control data with outbreak information, to support decision-makers and veterinary services in implementing and adapting the control measures needed to limit the spread of the infection. Given the emergency context, the Istituto Zooprofilattico Sperimentale dell’Abruzzo e del Molise “G. Caporale” (IZSAM), along with the National Reference Centre for the Study of Pestivirus and Asfivirus Diseases (CEREP) and the Italian General Directorate for Animal Health, developed a dedicated African Swine Fever Web-GIS dashboard (ASF Web-GIS dashboard). The platform was dynamically updated to meet evolving operational needs, drawing on data systematically recorded within the national information systems. The aim of this paper is to describe the ASF Web-GIS dashboard developed to monitor the spread of ASF, surveillance activities, and control measures across Italy, based on data collected from national information systems and other sources.

## 2. Materials and Methods

### 2.1. Dashboard Technology

The ASF Web-GIS dashboard aggregates information on surveillance, outbreaks, control measures, and zoning. It is based on Esri ArcGIS Dashboards, which is a tool integrated into the Esri ArcGIS Online Software as a Service (SaaS) platform, and enables users to convey information by presenting location-based analytics using interactive data visualisations on a single screen “https://www.esri.com/en-us/arcgis/products/arcgis-dashboards/overview” (accessed on 28 August 2026).

### 2.2. Data Sources

The data displayed in the ASF Web-GIS dashboard is stored in an Oracle Relational Database Management System (RDBMS). Surveillance data are collected, according to European Union requirements and Italian regulations, through the national information systems.

The National Animal Disease Information System (“Sistema Informativo Nazionale Malattie Animali”, SIMAN) was developed for the notification of outbreaks of animal infectious diseases, including ASF [25]. When an outbreak in domestic pigs or a case in wild boar is suspected by the veterinary services, the event is recorded in SIMAN as a suspected outbreak or case, respectively. Following diagnostic testing, the event is either confirmed or unconfirmed based on the test results of the sample. The epidemic event is considered closed once the outbreak or case has been contained. For SIMAN, surveillance data are available in the dashboard from the beginning of the ASF epidemic in January 2022.

The National Veterinary Information System for Food Safety (Sistema Informativo Nazionale Veterinario per la Sicurezza degli Alimenti, SINVSA) was adapted to collect data on ASF surveillance activities in both domestic pigs and wild boars. It records relevant sampling metadata and diagnostic test results. For the dashboard, data from SINVSA covering the most recent two years are extracted and displayed.

SIMAN and SINVSA include built-in consistency checks that prevent the registration of anomalies. For instance, SIMAN does not allow multiple outbreaks to be recorded at the same geographic coordinates, thereby preventing duplicate outbreak records. Similarly, SINVSA includes a check that prevents blood samples from being recorded when the domestic pig date of death has been entered.

SINVSA also includes the recording of enhanced surveillance activities (active searches) for wild boar carcasses. For each active search, the system records the date and duration of the search, the number of personnel involved, and whether dogs were used. Search activities are georeferenced to a specific 1 km × 1 km EEA reference grid for Europe (1 km). When carcasses are found, the number of wild boars detected is recorded and, when sampled, a corresponding sampling record is created with the specific sampling reason (“RICERCA ATTIVA”—active search). Consistency checks are performed by cross-referencing these records with corresponding samples, when available. The reported sampling reason is then verified to ensure consistency with enhanced surveillance activities. A number of carcasses greater than zero cannot be recorded unless a sampling record is provided. The layer displaying enhanced wild boar surveillance activities (active search) presents the data using the 1 km × 1 km EEA reference grid for Europe (1 km) “https://sdi.eea.europa.eu/catalogue/srv/api/records/d9d4684e-0a8d-496c-8be8-110f4b9465f6” (accessed on 28 August 2026).

The geographical layers of the restricted zones I, II and III, but also the CEV zone and UGC layers, are provided by technical staff of the Istituti Zooprofilattici Sperimentali or regional authorities and collected by IZSAM, upon request from the General Directorate for Animal Health of the Ministry of Health. The definition and updating of the restricted zones (restricted zones I, II and III) depend on the epidemiological evolution of the disease. The proposed zoning is submitted by the General Directorate for Animal Health to the European Commission for approval and, once approved, the corresponding geographical layers are transmitted to IZSAM for incorporation into the dashboard.

The layer of the physical barrier, depicting the updated status of the barriers’ establishment, is supplied by the General Directorate for Animal Health, based on information reported by the contractors appointed for the implementation of the barrier. Monthly, files containing updated information on the progress of the implementation status of motorway and non-motorway barriers as part of the African swine fever (ASF) spread prevention strategy are provided. This data undergoes manual quality checks by the data quality manager of the IZSAM to verify its completeness and consistency.

An overview of the data sources, types of data, available metadata, update frequency and basic data quality checks is provided in Table 1.

### 2.3. Data Flow

**Figure 2:**
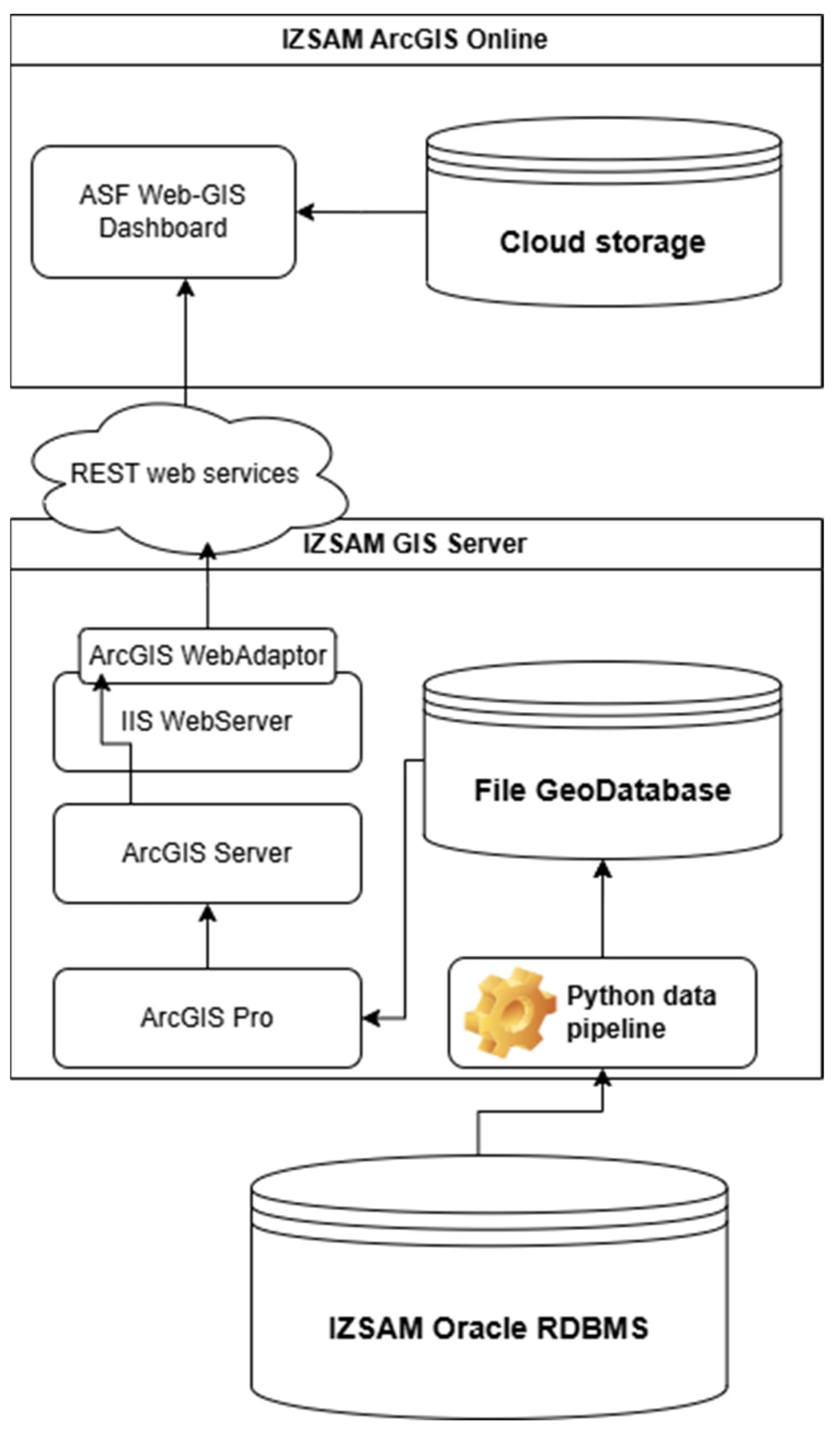
Architecture of the African Swine Fever (ASF) Web-GIS Dashboard data infrastructure. Rapidly changing epidemiological datasets are extracted daily from the Istituto Zooprofilattico Sperimentale dell’Abruzzo e del Molise (IZSAM) Oracle RDBMS through a scheduled Python data pipeline and stored in a read-only File GeoDatabase published as REST Feature Services via ArcGIS Server. Static or slowly changing datasets are instead maintained in ArcGIS Online cloud storage. The resulting hybrid architecture improves dashboard performance while avoiding replication of frequently updated operational data in the cloud.

A hybrid architecture, combining ArcGIS Online cloud resources with in-house GIS services (Figure 2), was designed to optimise the information processed and the performance of the ASF Web-GIS dashboard.

Within the ArcGIS Online environment, a cloud storage repository hosts static or slowly changing datasets, which do not require frequent updates. In parallel, a Python 3.14 data pipeline scheduled daily on a Windows Server retrieves rapidly changing datasets from Oracle sources, selects only the relevant information by removing unnecessary fields, and populates a read-only Esri File GeoDatabase “https://pro.arcgis.com/en/pro-app/latest/help/data/geodatabases/manage-file-gdb/file-geodatabases.htm” (accessed on 28 August 2026).

The File GeoDatabase is published as a multi-layer REST Feature Service “https://enterprise.arcgis.com/en/server/11.3/publish-services/windows/what-is-a-feature-service-.htm” (accessed on 28 August 2026) through an on-premises ArcGIS Server 11.3 exposed to stakeholders involved in emergency management (local, regional, and national veterinary services, Istituti Zooprofilattici Sperimentali staff, and the personnel of the General Directorate for Animal Health) using the ArcGIS Web Adaptor component deployed on an IIS Web Server.

This architecture avoids copying frequently updated operational data into the ArcGIS Online cloud environment, while still allowing the ASF Web-GIS Dashboard to be continuously powered with refreshed information. Feeding the REST Feature Service from a File GeoDatabase significantly improves data loading, navigation and query performance for end users.

Regarding usability, the dashboard was designed to provide easy access to the main surveillance information for its intended users. No formal usability assessment was conducted. The ASF Web-GIS dashboard is not publicly accessible, as it is intended to support ASF monitoring by Veterinary Services, staff of the Istituti Zooprofilattici Sperimentali, and the General Directorate for Animal Health of the Ministry of Health. Access is restricted to authorised users and is provided upon request.

## 3. Results

**Figure 3:**
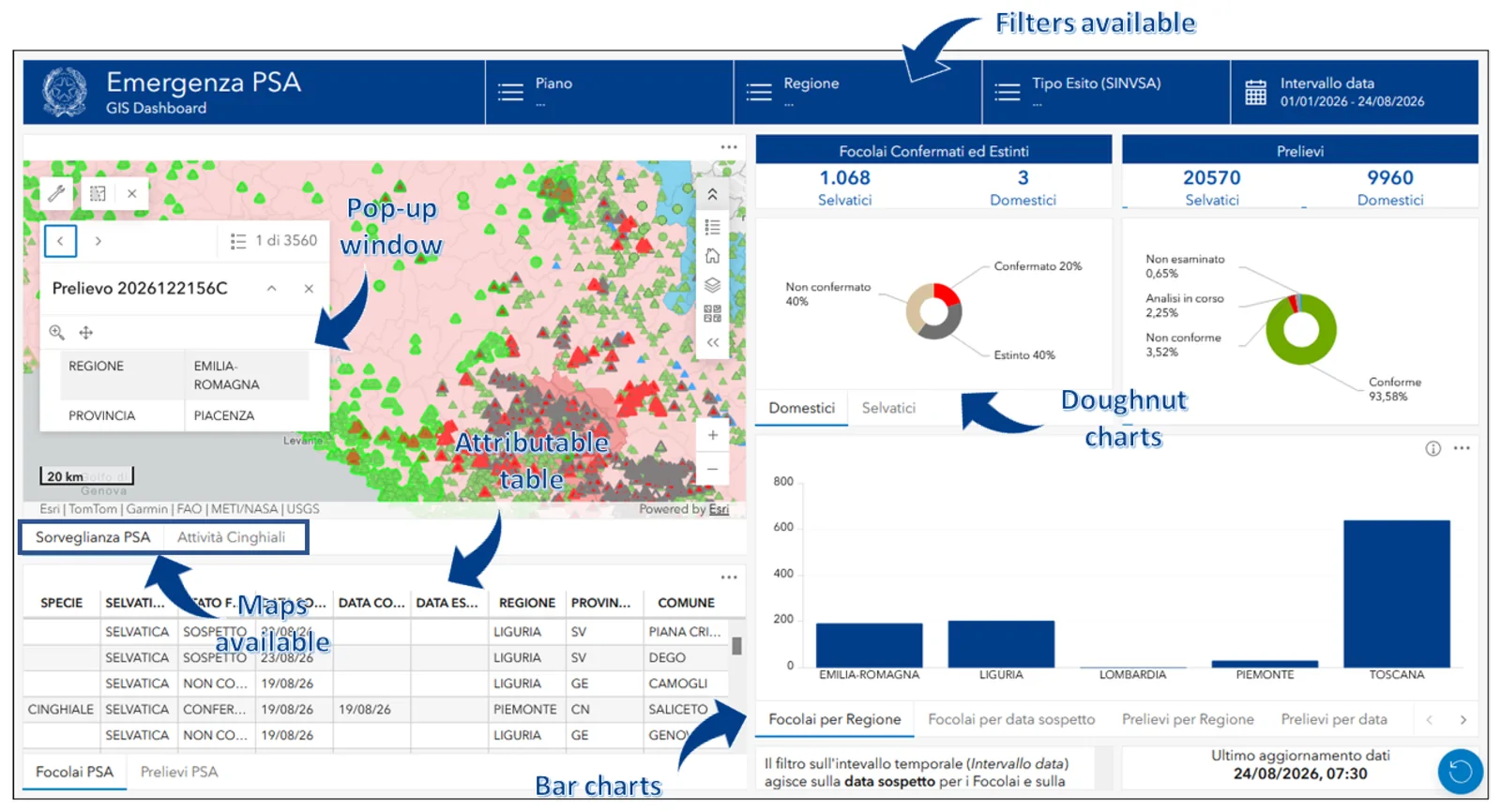
Example of a query available in the African Swine Fever (ASF) Web-GIS dashboard: The locations of ASF cases in wild boars are displayed on the map by clicking on the specific section of the pie chart; in addition, samples from the surveillance plan for wild boars can be viewed by filtering for that specific plan in the top tab. By clicking on a specific point on the map, it is possible to visualise the sample details. Source: ASF Web-GIS dashboard, accessed on 24 August 2026.

The interface of the ASF Web-GIS dashboard is designed to easily present surveillance data in the context of the measures undertaken (Figure 3).

Over the two-year sampling period displayed in the dashboard (the data reported refer to 19 August 2024–19 August 2026), 73,088 wild boar and 43,455 domestic pig samples have been collected by the National Veterinary Services, analysed for ASF by the Istituti Zooprofilattici Sperimentali and registered in SINVSA. Outbreak and case data are displayed from the beginning of the epidemic in January 2022 up to 19 August 2026, comprising 4282 cases in wild boars and 56 outbreaks in domestic pigs, including confirmed or closed outbreaks. In addition, the ASF Web-GIS dashboard displays 27 suspected and 2924 unconfirmed cases in wild boar and 167 unconfirmed outbreaks in domestic pigs. For the same period, the ASF Web-GIS dashboard also reports 82,458 active searches conducted across the national territory, along with the level of implementation of the closure of 627 motorway and non-motorway barrier gates.

Two maps are available in the ASF Web-GIS dashboard for consultation. The first one is related to ASF surveillance in Italy, while the second one shows the main details related to surveillance activities in wild boars, enabling the differentiation of the various surveillance and control activities carried out in the field (population reduction, detection of wild boar carcasses, active carcass search operations, and roadkill events).

The pop-up windows triggered by clicking on the map allow users to view all detailed information associated with the data recorded in the original information systems, both for sampling (unique identification number, sampling date, region, province, municipality, species, sex, preservation status, and laboratory test result) and domestic outbreaks or wildlife cases (status, region, province, municipality, confirmation date, suspicion date, closing date, species).

**Figure 4:**
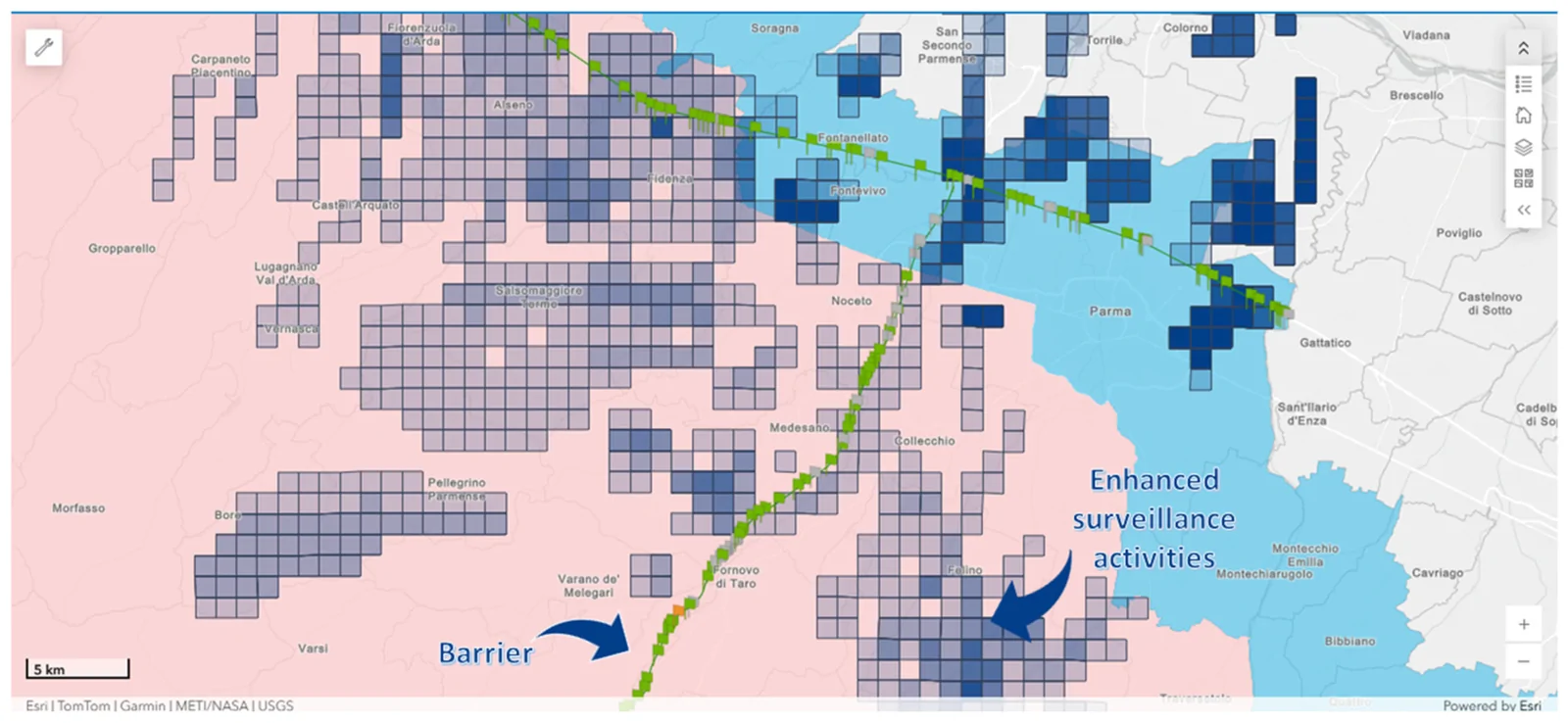
Layers displayed in the ASF Web-GIS dashboard, including the barrier layer used to visualise highway gate closures implemented to limit the spread of African Swine Fever (ASF), and the active searches layer representing monitoring activities on wild boar populations. Source: ASF Web-GIS dashboard, accessed on 24 August 2026.

In addition to the outbreak and specimens’ layers, which are characterised based on status and outcome, respectively, the following are available in the maps (Figure 4):

- The barrier layer that allows visualisation of highway gate closure activities conducted to limit the spread of African Swine Fever Virus;
- Layer related to active searches on wild boars shown as the 1 km × 1 km EEA reference grid for Europe (1 km);
- Layer of restricted zones as per Commission Implementing Regulation (EU) 2023/594 [17] and following amendments;
- Layer of the CEV Zone, the high-risk area for disease transmission;
- Layer of the UGC, areas of the territory implemented for the management of the wild boar species, as part of the wildlife hunting plan.

The ASF Web-GIS dashboard includes filtering options (by sampling plan (domestic or wild), region, time frame, and test results), thereby enhancing user interaction with the map and its spatial data. This allows for launching queries to the underlying database on the national surveillance plan implemented, the SINVSA, Italian regions, sampling results and dates of sampling.

Additionally, the ASF Web-GIS dashboard includes graphical and numerical information:

- Indicators: The number of cases in wild boars and outbreaks in domestic pigs, and the number of sampled animals by species.
- Charts: Two doughnut charts are provided in the dashboard. These charts show the number of confirmed and not confirmed, suspected cases or outbreaks, and the number of animals sampled by test results.
- Charts: Five bar charts report the number of outbreaks/cases by region and by month, the number of animals sampled by region and sampling point and by month and the number of 1 km × 1 km grid cells inspected during active carcass search operations.
- Tables: Two tables, one of which pertains to sampling, and the other to outbreaks in domestic animals and cases detected in wildlife, display detailed information corresponding to the data visualised on the map.

All data displayed in maps and graphs, as well as in tables, dynamically change according to the filter applied. Similarly, by interacting with the charts, it is possible to apply filters to the dataset that dynamically alter the visualization of information on the map.

The dashboard is actively used by Veterinary Services, staff of the Istituti Zooprofilattici Sperimentali, and the General Directorate for Animal Health for ASF disease monitoring. Since its implementation, its continued operational use has resulted in iterative requests for new functionalities, modifications and improvements, which have been progressively incorporated into the dashboard up to its current version. A video demonstrating the main features and functionality of the dashboard is provided in the Supplementary Materials (Video S1).

## 4. Discussion

Data visualisation has been widely applied across public health domains, including disease surveillance, prevention and control, with graphical and geospatial techniques to support evidence-based decision-making [26]. The use of Geographic Information System (GIS)-based solutions, particularly in the field of disease control, represents one of the most effective and engaging approaches for communicating complex information and supporting coordinated actions across multiple sectors involved in disease prevention and control. Numerous Web-GIS-based public health surveillance systems are currently operational worldwide and are routinely used to monitor diseases such as avian influenza, West Nile disease, dengue fever, and also antibiotic susceptibility [26,27]. Many of these systems serve as valuable tools for epidemic intelligence, which explains their increasing adoption. However, only a limited number operate at a national scale and integrate comprehensive official surveillance datasets enriched with metadata that can effectively support decision-making processes. In addition, epidemiological data are often aggregated and visualised using choropleth maps [26,27].

Notably, two Web GIS-based public health surveillance systems had previously been developed by IZSAM. The first is the Arbo-Zoonet Information System, which includes a Web-GIS dashboard designed to explore the spatial distribution of arboviral disease outbreaks using both official and unofficial data sources [28]. The second is a Web-GIS dashboard developed to monitor the distribution of wildlife diseases in the Abruzzo Region, based exclusively on diagnostic results from wildlife samples tested by IZSAM laboratories [29].

Compared with existing platforms, the ASF Web-GIS dashboard is the first system implemented in Italy to support the collection and visualization of surveillance data for ASF, in addition to outbreak and case data, at the livestock–wildlife interface.

Data are updated daily through automated Python pipelines running on a local Windows Server and are made available to authorised users through a GIS dashboard developed using Esri Software-as-a-Service (SaaS) technology within a private cloud environment (ArcGIS Online). Through interactive maps, charts, tables, and key performance indicators (KPIs), the dashboard facilitates data analysis, supports decision-making processes, and may provide valuable support for planning and implementing measures aimed at controlling the spread of the disease.

The ASF Web- GIS dashboard provides access to data on surveillance activities, wild boar cases, domestic pig outbreaks, zoning measures, and other disease control interventions. The data displayed in the dashboard are collected through national information systems by Local and Regional Veterinary Services and the Istituti Zooprofilattici Sperimentali, under the coordination of the General Directorate for Animal Health and CEREP. In practice, the ASF Web-GIS dashboard is used daily by the General Directorate for Animal Health of the Ministry of Health and other decision-makers to monitor near-real-time information and trends related to the ASF emergency. In particular, in the event of a new confirmed case in wild boars or an outbreak in domestic pigs in an ASF-free area, the ASF Web-GIS Dashboard can support a basic assessment of the epidemiological situation. Using the basic Esri ArcGIS Dashboards tools available within the dashboard, users can measure the distance between positive samples or between a case or outbreak and the boundary of a restricted zone. This information may contribute to the assessment of proposals for the establishment or modification of restriction zones submitted to the European Commission. In addition, visualisation of surveillance data, including sampling and active search activities, may help local veterinary authorities identify areas where additional surveillance activities could be considered.

The daily update schedule enables near-real-time visualisation of surveillance data. No specific feedback indicating that the daily update frequency represents a limitation has been received from end users. Indeed, according to the available literature, only a few Web-GIS surveillance systems provide daily or real-time updates [27]. Moreover, if required, the update frequency could be further increased. The current configuration, however, helps limit the volume of data processed and transferred and supports efficient dashboard performance. For the same reason, a two-year temporal window was applied to ASF surveillance sampling data from SINVSA. This restriction does not apply to SIMAN data, for which records from the beginning of the epidemic are retained to support the longitudinal visualisation of notified ASF cases and outbreaks. Further limitations include the lack of a formal usability assessment; therefore, user satisfaction and ease of use were not quantitatively evaluated. Nevertheless, the continued operational use of the dashboard and the iterative development driven by the General Directorate for Animal Health of the Ministry of Health requirements provide practical evidence of its use in routine ASF disease monitoring, although these interactions do not constitute a formal usability assessment. Technical performance indicators, such as data-transfer reliability, update time and dashboard loading time, were not systematically evaluated. In addition, the practical impact of the dashboard on decision-making and disease-control outcomes was not formally assessed.

The ASF Web-GIS dashboard is primarily designed for data visualisation and does not currently provide direct access to downloadable datasets. Where access to the underlying data is required, these can be retrieved from the respective source information systems.

## 5. Conclusions

Given the large volume of data collected from wild boar and domestic pig populations during the ASF epidemic in Italy, the processes implemented within the Web-GIS dashboard represent a valuable example of how surveillance data, including wildlife data, can be effectively organised and made accessible to decision-makers. This has the potential to support the implementation of control measures for severe transboundary animal diseases such as African swine fever, for which timely interventions are important.

## Figures and Tables

**Table 1:** The table summarises the data sources contributing to the dynamic content of the ASF Web-GIS dashboard and subject to periodic updates. Static reference layers are not included.

Data Source	Type of Data	Data Collected	Update Frequency	Quality Checks
SIMAN	ASF outbreak and case data in domestic pigs and wild boar	Region, province, municipality, outbreak number, farm code, geographical coordinates, species involved in the outbreak, type of outbreak (diagnostic positivity, clinical outbreak, post-mortem findings), suspicion date, confirmation date, closing date	Daily	Completeness and consistency checks performed at source-system level
SINVSA	Data on sampling and enhanced passive surveillance activity	Domestic pig sampling plan: sampling strategy (sampling based on suspicion, surveillance plan, pre-moving inspection), sampling location (geographic coordinates), collection date, sample collected (material, species), additional information (animal identification, date of death, age, sex) Wild boar sampling plan: sampling strategy (sampling based on suspicion, surveillance plan), reason for sampling (“RITROVAMENTO SU SEGNALAZIONE”—found following a report, “RICERCA ATTIVA”—active search, “ABBATTUTI/CACCIATI”—killed/hunted), sampling location (geographic coordinates), collection date, sample collected (material, species), additional information (age, sex, animal involved in an accident, condition of the carcass, method of hunting) Active searches: the date and duration of the search, the number of personnel involved, whether dogs were used, the 1 km × 1 km grid cell code, number of carcasses and sampling record	Daily	Completeness and consistency checks performed at source-system level
General Directorate for Animal Health of the Ministry of Health	Zooning shapefile (Restricted zones I, II and III)	Restricted zone classification, restricted zone start date	It depends on the evolution of the epidemiological situation.	No quality check is needed
General Directorate for Animal Health of the Ministry of Health	Geographical locations of ongoing barrier installations	Motorway, geographic coordinates of the closure point, planned closure date, actual closure date	Monthly	Completeness and consistency checks performed on the file provided

## Data Availability

The original contributions presented in this study are included in the article/Supplementary Material. Further inquiries can be directed to the corresponding authors.

## Supplementary Materials

The following supporting information can be downloaded at: https://www.mdpi.com/article/10.3390/pathogens15090925/s1, Video S1: Dashboard Overview.

## Abbreviations

The following abbreviations are used in this manuscript:

ASF	African Swine Fever
CEREP	National Reference Centre for the Study of *Pestivirus* and *Asfivirus* Diseases
CEV	Viral Expansion Control Zone
GIS	Geographic Information System
IZSAM	Istituto Zooprofilattico Sperimentale dell’Abruzzo e del Molise “G. Caporale”
